# *Penthorum chinense* Pursh extract ameliorates hepatic steatosis by suppressing pyroptosis via the NLRP3/Caspase‐1/GSDMD pathway

**DOI:** 10.1002/fsn3.4165

**Published:** 2024-04-16

**Authors:** Ruixi Luo, Yudie Hu, La Wang, Zunli Ke, Wenjia Wang, Ping Wang, Weiyi Tian

**Affiliations:** ^1^ Department of Immunology and Microbiology School of Basic Medical Sciences, Guizhou University of Traditional Chinese Medicine Guiyang China

**Keywords:** lipotoxicity, nonalcoholic fatty liver disease, *Penthorum chinense* Pursh, Pyroptosis

## Abstract

The primary catalyst for nonalcoholic fatty liver disease (NAFLD) is widely recognized as the induction of lipotoxicity in hepatocytes by an excess of fatty acids. In China, *Penthorum chinense* Pursh (PcP) is commonly employed as a functional food due to its known hepatoprotective properties. The present study aimed to investigate the influence of PcP extract on in vivo and in vitro models of NAFLD. We found that PcP extract can attenuate palmitic acid (PA)‐induced lipotoxicity in HepG2 cells. PA was observed to trigger pyroptosis, as indicated by the increased expression of NLRP3 and GSDMD/N, activation of Caspase‐1, and subsequent release of IL‐1β and IL‐18. However, these changes were reversed after PcP was administered. Furthermore, the application of an NLRP3 agonist inhibited the protective effects of PcP on lipotoxicity, indicating that PcP decreased lipotoxicity by inhibiting the NLRP3/Caspase‐1/GSDMD pathway. Ultimately, we established a rat model of NAFLD through the administration of a high‐fat diet (HFD), followed by the oral delivery of PcP extracts. The results demonstrated that the administration of PcP extract effectively decreased dyslipidemia and hepatic steatosis, which coincided with a decrease in hepatic pyroptosis through modulation of the NLRP3/Caspase‐1/GSDMD pathway in liver tissues. Overall, our findings provide insight into the mechanism by which PcP extracts alleviate hepatic steatosis, highlighting the potential significance of modulating the NLRP3/Caspase‐1/GSDMD pathway in the context of pyroptosis.

## Graphical abstract

Administration of PcP extract effectively alleviates hepatic steatosis by inhibiting pyroptosis both in vivo and in vitro. Modulating the NLRP3/Caspase‐1/GSDMD pathway might be a crucial mechanism underlying the therapeutic effects of PcP.

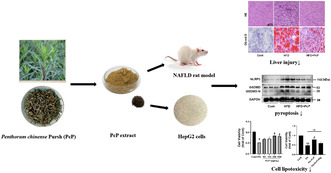

## 1 INTRODUCTION

Nonalcoholic fatty liver disease (NAFLD) is a prevalent and progressively recognized liver disorder marked by the excessive build‐up of lipids within hepatocytes. The increasing prevalence of NAFLD has raised concerns within the public health domain, as the disease has the potential to advance to more severe liver conditions (Li, Spiers, & Brindley, 2022). Regrettably, no effective pharmaceutical interventions are currently available for the treatment of NAFLD.

The development of NAFLD is a complex and multifaceted process and involves diverse factors, including insulin resistance, oxidative stress, inflammation, and changes in the gut microbiota (Chi, 2017). Nevertheless, the primary factor that initiates and advances the early stages of NAFLD is the surplus build‐up of fatty acids within liver cells, which leads to lipotoxicity (Cusi, 2012). This excessive accumulation of fatty acids detrimentally affects hepatocyte function, ultimately culminating in cell death. Our previous study indicated that pyroptosis was the predominant mechanism of cell death during the progression of NAFLD (Zeng et al., 2020). Moreover, the NLRP3/Caspase‐1/GSDMD pathway is a critical signaling cascade involved in pyroptosis (Zeng et al., 2020). Prolonged exposure to stress induced by excess fatty acids can upregulate the NLRP3 inflammasome complex, resulting in the cleavage of GSDMD. Consequently, the ability to modulate pyroptosis has significant implications for the development of therapeutic strategies for NAFLD (Li et al., 2023).

*Penthorum chinense* Pursh (PcP), a perennial herbaceous plant classified under the family Saxifragaceae, is commonly referred to as “Chinese Penthorum”. In China, the plant is recognized as a functional food due to its hepatoprotective properties (Nabi et al., 2023). PcP has been extensively utilized for its potential medicinal properties, including its anti‐inflammatory, antioxidative, and detoxifying effects (Wang et al., 2020). Notably, Gansu granules, a Chinese patent medicine derived from PcP extracts, are presently utilized in clinical settings for managing liver disease (Wang et al., 2015). Currently, numerous studies have documented the potential of PcP to improve NAFLD both in vivo and in vitro (Guo et al., 2018; Li, Zhao, et al., 2022). However, comprehensive knowledge on the underlying mechanism remains elusive. Our previous research indicated that inhibiting pyroptosis helps ameliorate NAFLD in rats; thus, we sought to investigate whether PcP can alleviate NAFLD by modulating pyroptosis‐related pathways.

## 2 MATERIALS AND METHODS

### 2.1 Preparation of PcP reagents

A dry fine powder of PcP aqueous extract was purchased from Baicao Organism Biotech (BC10125, Chengdu, China). Subsequently, the PcP powder was dissolved in sterile deionized water and filtered (331,001, NEST Biotechnology, China). The resulting filtrate was further diluted to a concentration of 100 mg/mL and stored at −20°C for subsequent in vitro experiments.

### 2.2 LC–MS/MS analysis

PcP reagent was dissolved and centrifuged at 12,000 rpm. The supernatant obtained from the centrifugation was then utilized for LC–MS/MS analysis. UHPLC was conducted using a Thermo Scientific UltiMate 3000 HPLC system (USA) and a Sepax GP‐C18 column (1.8 μm, 2.1 mm × 150 mm). The UHPLC system utilized 0.1% formic acid in water as phase A and 100% ACN as phase B, with a flow rate of 0.3 mL/min. Mass spectrometry analysis was conducted in both positive and negative modes. The obtained data were processed using MS‐DIAL (version 4.7).

### 2.3 Cell culture and treatment

HepG2 cells (CL‐0103, Procell Life Science & Technology, China) were cultured in DMEM (BC‐M‐005, BioChannel, China) supplemented with 10% FBS (C2910‐0500, VivaCell, China) under standard conditions (37°C, 5% CO_2_). In the in vitro experiments, cells were stimulated with palmitic acid (400 μM, PA, Aladdin, China) to induce lipotoxicity as previously reported (Luo et al., 2020). In the PcP group, the mother liquor of the PcP extract was added and diluted in culture medium to the indicated concentration. Nigericin (T3092, TargetMol, USA) was added to the wells as an NLRP3 activator.

### 2.4 Cell viability

Briefly, cells were counted and seeded into a 96‐well plate (1014000, SAINING, China) with or without the presence of PA or PcP. After the indicated times, new medium supplemented with 10% CCK8 solution (M4839, AbMole, China) was added. The absorbance was read at 450 nm 2–4 h later with a microplate reader.

### 2.5 TG measurement and oil red O staining

After the indicated treatments, the cells or liver sections were subjected to staining with oil red O Solution (DL0008 or DL0001, Leagene Biotechnology Co., Ltd., China). The TG content in the cells was quantified using a triglyceride quantification kit (MAK266, Sigma–Aldrich, USA).

### 2.6 Transmission electron microscopy (TEM)

A549 cells were prefixed and embedded. After the indicated treatments, the sections were observed under a transmission electron microscope (JEM‐1400, Japan).

### 2.7 Caspase‐1 activity

A Caspase‐1 Assay Kit (KTA3020, Abbkine, China) was used to detect caspase‐1 activity. The absorbance at 405 nm was measured 1–2 h later using a microplate reader. Caspase‐1 activity was evaluated as enzyme units (U/mg prot).

### 2.8 Elisa

ELISA kits were used to assess IL‐1β (RK00009, ABclonal, China) and IL‐18 (JL20882, Jianglai bio, China) levels in rat serum, as well as IL‐1β (SEKH‐0002, Solarbio, China), IL‐18 (U96‐1964E, YOBIBIO, China), IL‐6 (ELK1156, ELK Biotechnology, China) and TNF‐α (ab285312, Abcam, USA) levels in HepG2 cell culture medium.

### 2.9 Immunofluorescence

After the indicated treatment, the cells were fixed and incubated with antibodies, and the cells were visualized using a fluorescence microscope. The specific antibodies and DAPI used in this study were as follows: NLRP3 (1:500, ET‐1610‐93, Huabio, China), Gasdermin D (1:300, GSDMD, 66387–1, Proteintech, China), 4′,6‐diamidino‐2‐phenylindole (DAPI, GTX16206, GeneTex, USA), phospho‐NF‐κB p65 (Ser536) (1:200, p‐p65, AF2006, Affinity, China), HyperFluor 488 goat anti‐rabbit IgG (H + L) antibody (1:1000, BF03008, Biodragon, China), and goat anti‐mouse IgG‐PE antibody (1:500, abs20007, Absin, China).

### 2.10 Q‐PCR

Total RNA Extraction Reagent (RE600, Coolaber, China) and a Magic 1st cDNA Synthesis Kit (M211, Magic‐bio, China) were used to extract and reverse transcribe the RNA to cDNA. Q‐PCR was performed with StarLighter SYBR Green (FS‐Q1002, Beijing Foreverstar Biotech, China) as previously described. The primer information is provided in Table S1. β‐Actin primer pair (KGDN17, KeyGEN, China) was purchased and used as the reference gene.

### 2.11 Western blot

Following the specified treatment, the cells were lysed using RIPA buffer (E‐BC‐R327, Elabscience, China). The protein content was assessed through BCA protein assays (G0418W, Geruisi‐bio, China). Subsequently, the proteins were separated and transferred to a PVDF membrane. After blocking for 1 h, the membrane was incubated with antibodies. The blots were visualized using Super ECL Plus (MA0186, MeilunBio, China) with a Bio‐Rad ChemiDoc Touch Imaging System. The antibodies used were as follows: NLRP3 (1:1000, ET‐1610‐93, Huabio, China), GSDMD (1:1000, R24514, Zen‐bio, China), GSDMD, N‐terminal (1:1000, GSDMD‐N, DF12275 and DF13758, Affinity Biosciences, China), GAPDH (1:50000, bs‐0755R, Bioss, China), goat anti‐rabbit IgG secondary antibody (1:5000, L3012, Signalway Antibody, China), and goat anti‐mouse IgG secondary antibody (1:3000, FNab09848, Fine Test, China).

### 2.12 Animal experiments

Twenty‐eight SD rats (180–200 g) were procured from Dashuo Experimental Animal Co., Ltd. [SCXK (Chuan) 2020–030, Chengdu, Sichuan, China]. After a one‐week adaptation period, the rats were randomly divided into the following groups: a chow diet group (control, *n* = 11) and a high‐fat diet (HFD) group (*n* = 17). Rats in the HFD group were provided an HFD (7.6% standard diet, 10% lard, 5% yolk powder, 5% sucrose, 2% cholesterol, 0.2% sodium cholate, and 0.2% propylthiouracil) obtained from Dashuo Experimental Animal Co., Ltd., for 8 weeks. Subsequently, five rats from each group were euthanized to establish the NAFLD model. The remaining 12 rats from the HFD group were subsequently randomized into two groups: the HFD group (*n* = 6) and the HFD + PcP group (*n* = 6). PcP powder was dissolved in sterile deionized water and administered orally via gavage once daily for 4 weeks at a dosage of 3 g/kg/BW. Conversely, the rats in the HFD group were administered an equivalent volume of water. Subsequently, the rats were euthanized, and liver and serum samples were collected for further analysis.

### 2.13 Level of TG and TC in the liver

A triglyceride assay kit (A110‐1‐1, NJJCBIO, China) and a total cholesterol assay kit (A111‐1‐1, NJJCBIO, China) were used to measure the TG and TC contents in the liver.

### 2.14 Hematoxylin and eosin (HE) staining

Liver tissue samples were fixed and cut into thin sections. These sections were then subjected to deparaffinization and rehydration. Next, the sections were immersed in hematoxylin and eosin solution to stain the nuclei and cytoplasm as previously reported (Luo et al., 2020). Finally, the sections were examined under a light microscope.

### 2.15 Immunohistochemistry

Immunohistochemical staining was conducted using antibodies targeting NLRP3 and GSDMD to visualize the protein expression in liver tissues. The tissues were fixed and subsequently sectioned into slices. Following incubation with antibodies, the slices were observed.

### 2.16 Statistical analysis

Statistical analyses were performed using SPSS statistical software. The data are presented as the mean ± standard deviation (SD). Group differences were assessed using one‐way ANOVA followed by Duncan's multiple‐range test. Statistical significance was defined as *p* < .05.

## 3 RESULTS

### 3.1 Component of PcP extract

The LC–MS/MS results revealed approximately 307 constituents in the PcP extract (Table S2). The PcP extract contained substantial quantities of polyphenols, including flavonoids, phenolic acids, and other compounds, which was consistent with the results of prior studies (Guo et al., 2015).

### 3.2 PcP extract mitigated PA‐induced damage in HepG2 cells

**FIGURE 1:**
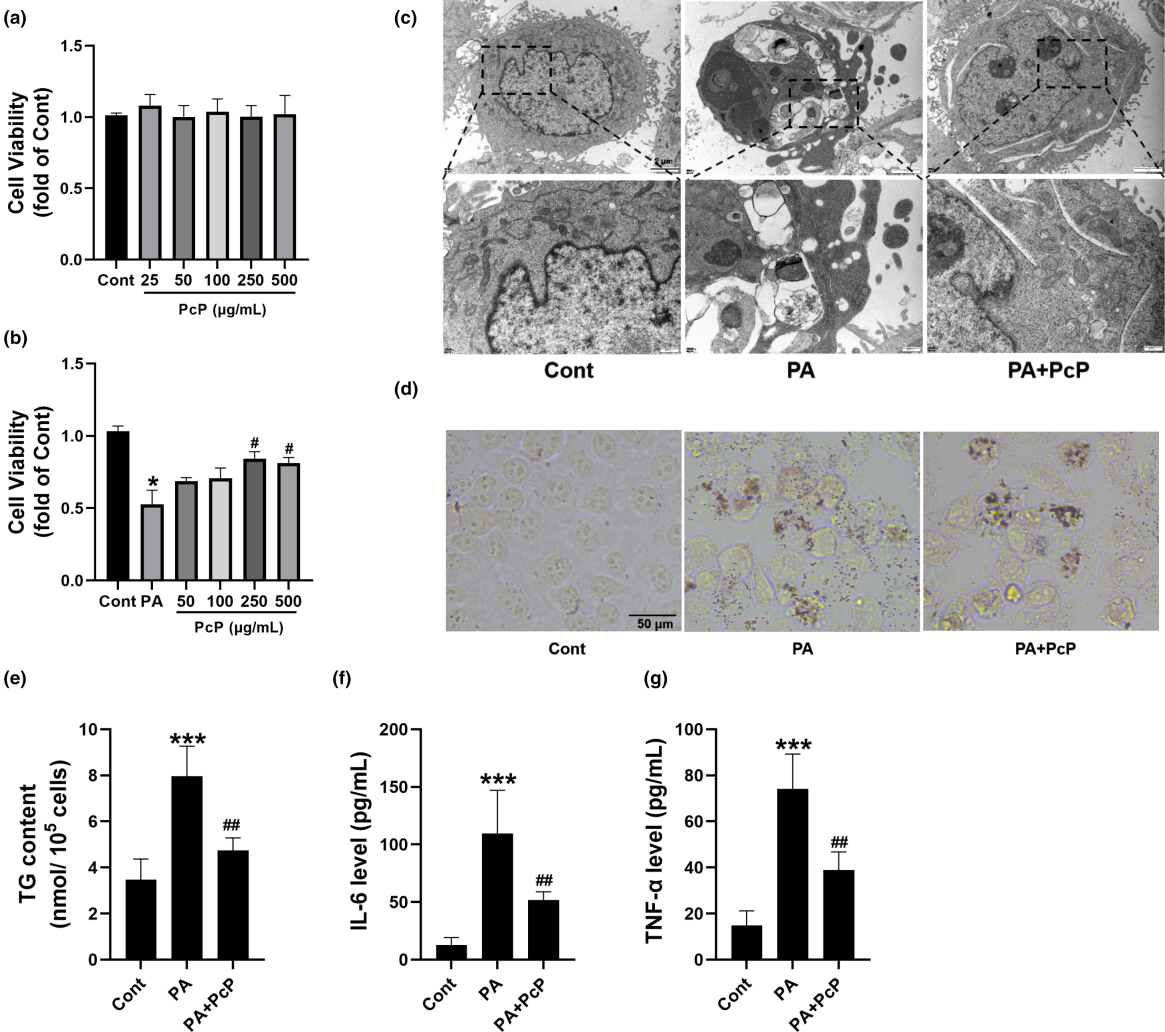
PcP extract decreased PA‐induced damage in HepG2 cells. (a) HepG2 cells were treated with different concentrations (25, 50, 100, 250, and 500 μg/mL) of PcP, and cell viability was analyzed by a CCK‐8 assay. (b) HepG2 cells were treated with PA (400 μM) with or without different doses (50, 100, 250, and 500 μg/mL) of PcP, and cell viability was analyzed by a CCK‐8 assay. (c) HepG2 cells were treated with PA (400 μM) with or without 250 μg/mL PcP. The cell morphology was observed by TEM. Scale bar, 2 μm. (d) Oil red o staining was performed to observe lipid deposition. (e) The TG content of cells in different groups was measured using a triglyceride quantification kit. (f) and (g) IL‐6 and TNF‐α levels in the cell medium were determined by ELISA. All experiments were repeated at least three times independently. The data are expressed as the mean ± SD (*p < .05, ***p < .01 vs. the Cont group; ^#^ *p* < .05, ^#*#*^ *p* < .01 vs. the PA group).

The cytotoxicity of PcP extract on HepG2 cells was initially assessed. The results showed that the PcP extract had no cytotoxic effect at concentrations up to 500 μg/mL (Figure 1a). We then established a model of PA (400 μM)‐induced lipotoxicity in HepG2 cells as previously reported (Li et al., 2021). Then, the cells were treated with or without PcP extract (50, 100, 250, or 500 μg/mL). Notably, cell viability decreased to 53% following PA treatment, whereas cell viability was restored following administration of the PcP extract at concentrations of 250 and 500 μg/mL (Figure 1b). For subsequent experiments, PcP extract was administered at a concentration of 250 μg/mL. TEM showed that after PA treatment, the cells exhibited morphologies that typically indicate death, including cell shrinkage, chromatin condensation, cell membrane blebbing, and rupture. However, the degree of cell death was alleviated after PcP treatment (Figure 1c). Moreover, PA‐induced significant accumulation of lipid droplets within the cytoplasm, whereas the presence of PcP mitigated this response (Figure 1d). Furthermore, we detected an increase in TG content following PA treatment, while a decrease in TG content was detected in the cells of the PcP group (Figure 1e). In addition, the concentrations of inflammatory factors in the cell medium were assessed. Following treatment with PA, the cells exhibited increased secretion of TNF‐α and IL‐6, which was subsequently decreased by supplementation with PcP (Figure 1f,g). The above results indicated that PcP extract effectively mitigated PA‐induced damage in HepG2 cells.

### 3.3 PcP extract decreased PA‐induced pyroptosis in HepG2 cells

**FIGURE 2:**
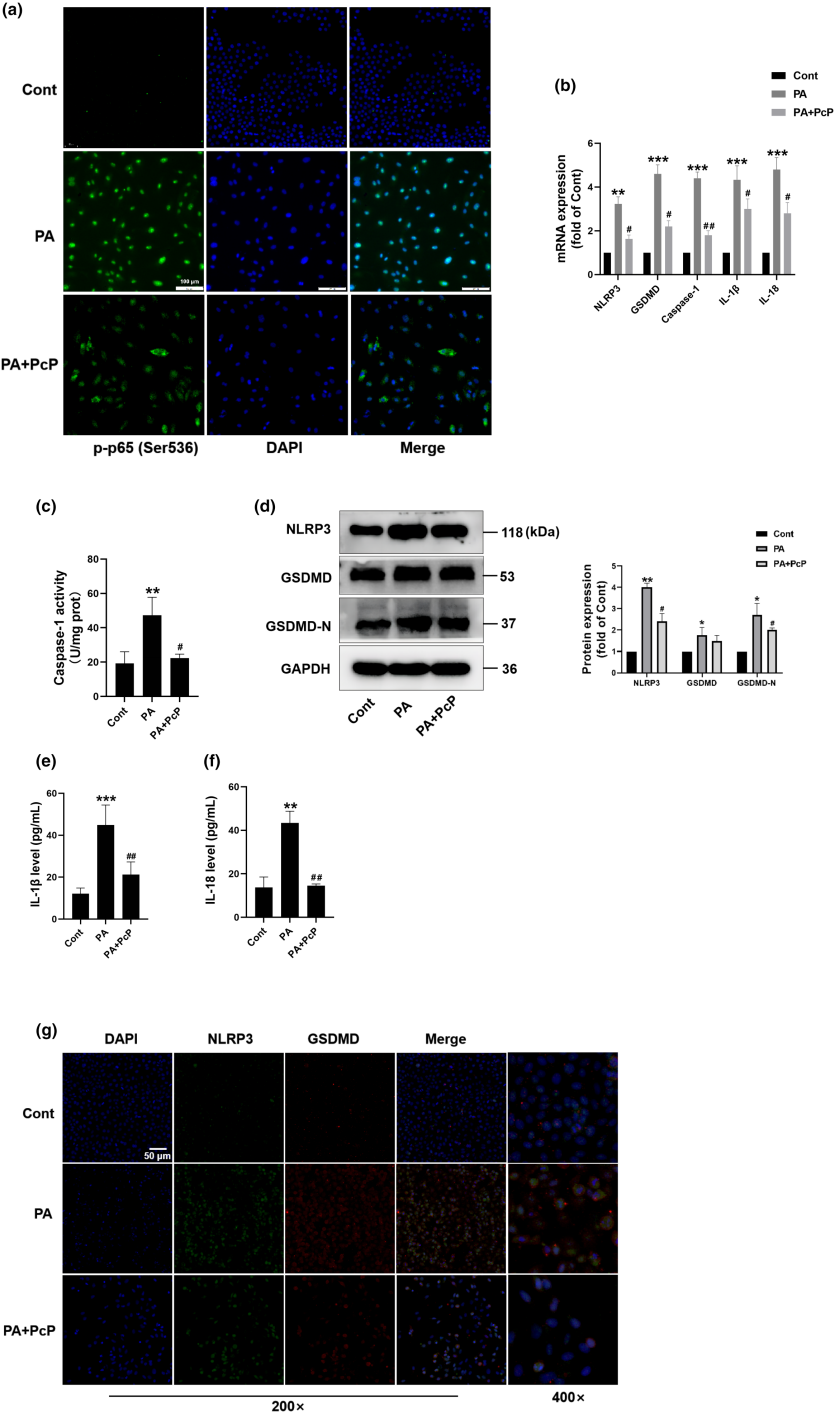
PcP extract decreased PA‐induced pyroptosis in HepG2 cells. HepG2 cells were treated with PA (400 μM) with or without 250 μg/mL PcP. (a) Representative images of immunofluorescence staining of phospho‐NF‐kB p65 (Ser536) in HepG2 cells in different groups. Scale bar, 100 μm. (b) The mRNA levels of *NLRP3*, *Caspase‐1*, *GSDMD*, *IL‐1β*, and *IL‐18* were detected by Q‐PCR. (c) The caspase‐1 activity of cells in different groups was measured by a caspase‐1 assay kit. (d) The protein levels of Nlrp3 and GSDMD/‐N were detected by Western blotting. (e, f) The protein levels of IL‐1β and IL‐18 in the cell medium were measured by ELISA. (g) Representative images of immunofluorescence staining of NLRP3 and GSDMD in HepG2 cells in different groups. Scale bar, 50 μm. All experiments were repeated at least three times independently. The data are expressed as the mean ± SD (*p < .05, **p < .05, ***p < .01 vs. the Cont group; ^#^ *p* < .05, ^#*#*^ *p* < .01 vs. the PA group).

Our previous study indicated that exposure to PA led to the induction of pyroptosis in HepG2 cells, and inhibiting pyroptosis was found to alleviate lipotoxicity (Zeng et al., 2020). Therefore, we evaluated whether PcP extract could effectively inhibit pyroptosis in HepG2 cells. We first assessed the activation of NLRP3 upstream of the NF‐κB pathway. Immunofluorescence showed that after PA induction, phospho‐NF‐κB p65 (p‐p65, Ser536) was activated and translocated into the nucleus, indicating that the NF‐κB pathway was activated. However, PCP treatment reversed these changes (Figure 2a). In comparison to the PAs, the PcP extract notably decreased several crucial genes associated with pyroptosis, namely, *IL‐1β*, *IL‐18*, *NLRP3*, *GSDMD*, and *Caspase‐1*, based on the Q‐PCR results (Figure 2b). Moreover, the PcP extract reduced caspase‐1 activity, which was elevated in the PA group (Figure 2c). Moreover, Western blot analysis indicated that NLRP3 and GSDMD/‐N were significantly upregulated after treatment with PA. However, the administration of PcP extract effectively reversed these alterations (Figure 2d). Additionally, we found that the cells released increased levels of IL‐1β and IL‐18 upon PA treatment, which was effectively attenuated by the application of PcP extract (Figure 2e,f). Furthermore, immunofluorescence staining analysis indicated that the levels of NLRP3 and GSDMD were increased following PA treatment, while this increase was suppressed by the administration of PcP extract (Figure 2g). The above results suggested that PcP extract decreased PA‐induced pyroptosis in HepG2 cells.

### 3.4 PcP extract decreased PA‐induced lipotoxicity by inhibiting the NLRP3/Caspase‐1/GSDMD pathway in HepG2 cells

**FIGURE 3:**
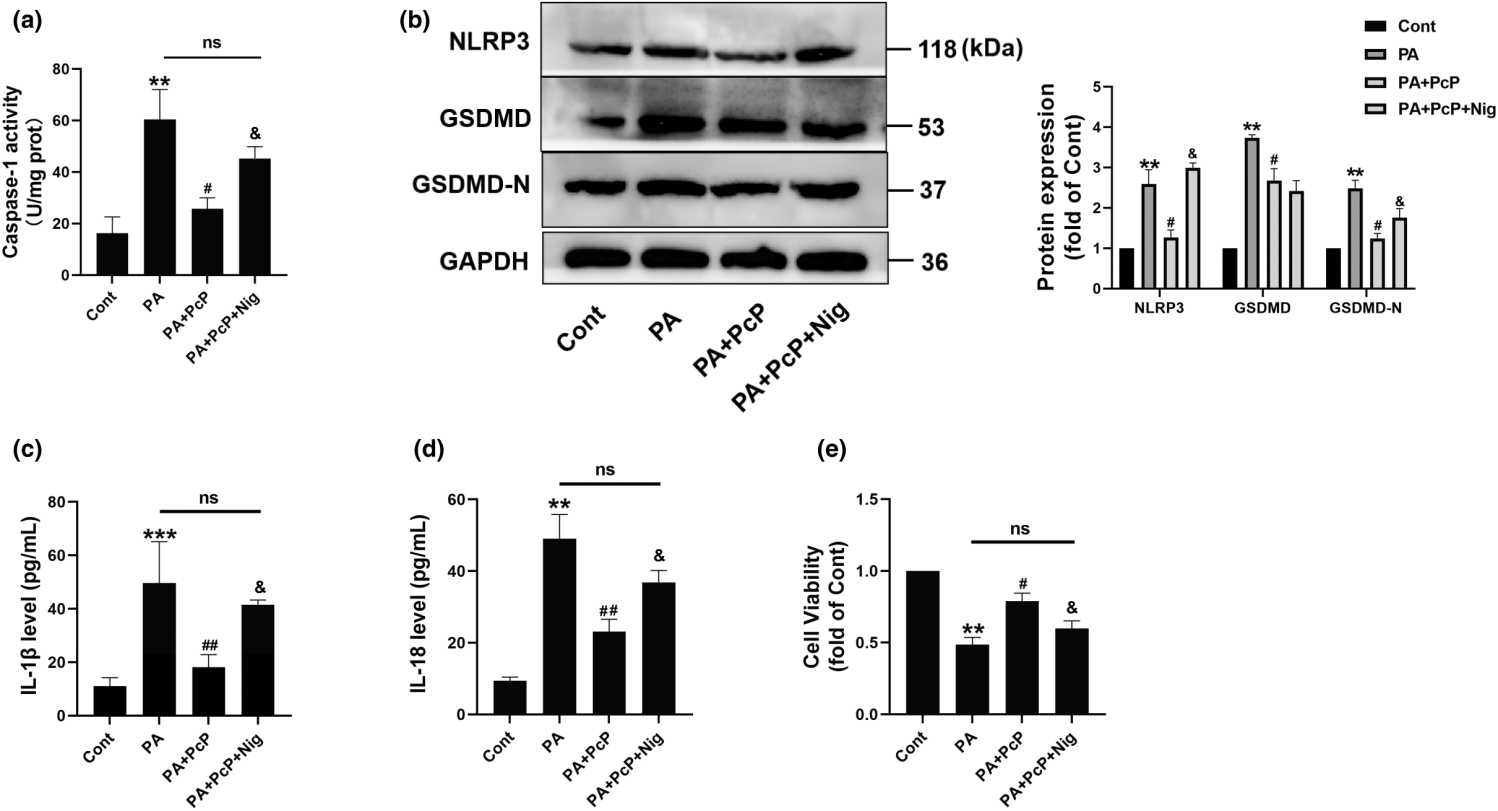
PcP extract decreased PA‐induced lipotoxicity by inhibiting the NLRP3/Caspase‐1/GSDMD pathway in HepG2 cells. (a) HepG2 cells were treated with PA (400 μM) with or without 250 μg/mL PcP or 10 μM nigericin, and the caspase‐1 activity of cells in different groups was measured with a caspase‐1 assay kit. (b) The protein levels of Nlrp3 and GSDMD/‐N were detected by Western blotting. (c, d) The protein levels of IL‐1β and IL‐18 in the cell medium were measured by ELISA. (e) Cell viability was analyzed by a CCK‐8 assay. All experiments were repeated at least three times independently. The data are expressed as the mean ± SD (**p < .05, ***p < .01 vs. the Cont group; ^#^ *p* < .05, ^#*#*^ *p* < .01 vs. the PA group; ^&^ *p* < .05 vs. the PA + PcP group).

As the regulation of pyroptosis is crucially influenced by the NLRP3/Caspase‐1/GSDMD pathway, we used nigericin, an activator of NLRP3. Consistent with previous findings, the PcP extract suppressed PA‐induced pyroptosis, as indicated by a decrease in caspase‐1 activity, decreased levels of the NLRP3 and GSDMD/N proteins, and reduced secretion of IL‐1β and IL‐18. The ability of the PcP extract to inhibit pyroptosis was notably impaired by the inclusion of nigericin in the experiment (Figure 3a–d). Furthermore, the introduction of nigericin counteracted the impact of PcP on cell viability (Figure 3e), suggesting a diminished protective effect of PcP against lipotoxicity. Consequently, based on the aforementioned results, the primary mechanism through which PcP extract reduces pyroptosis involves inhibition of the NLRP3‐related pathway.

### 3.5 PcP extract attenuated dyslipidemia and hepatic injury in HFD‐fed rats

**FIGURE 4:**
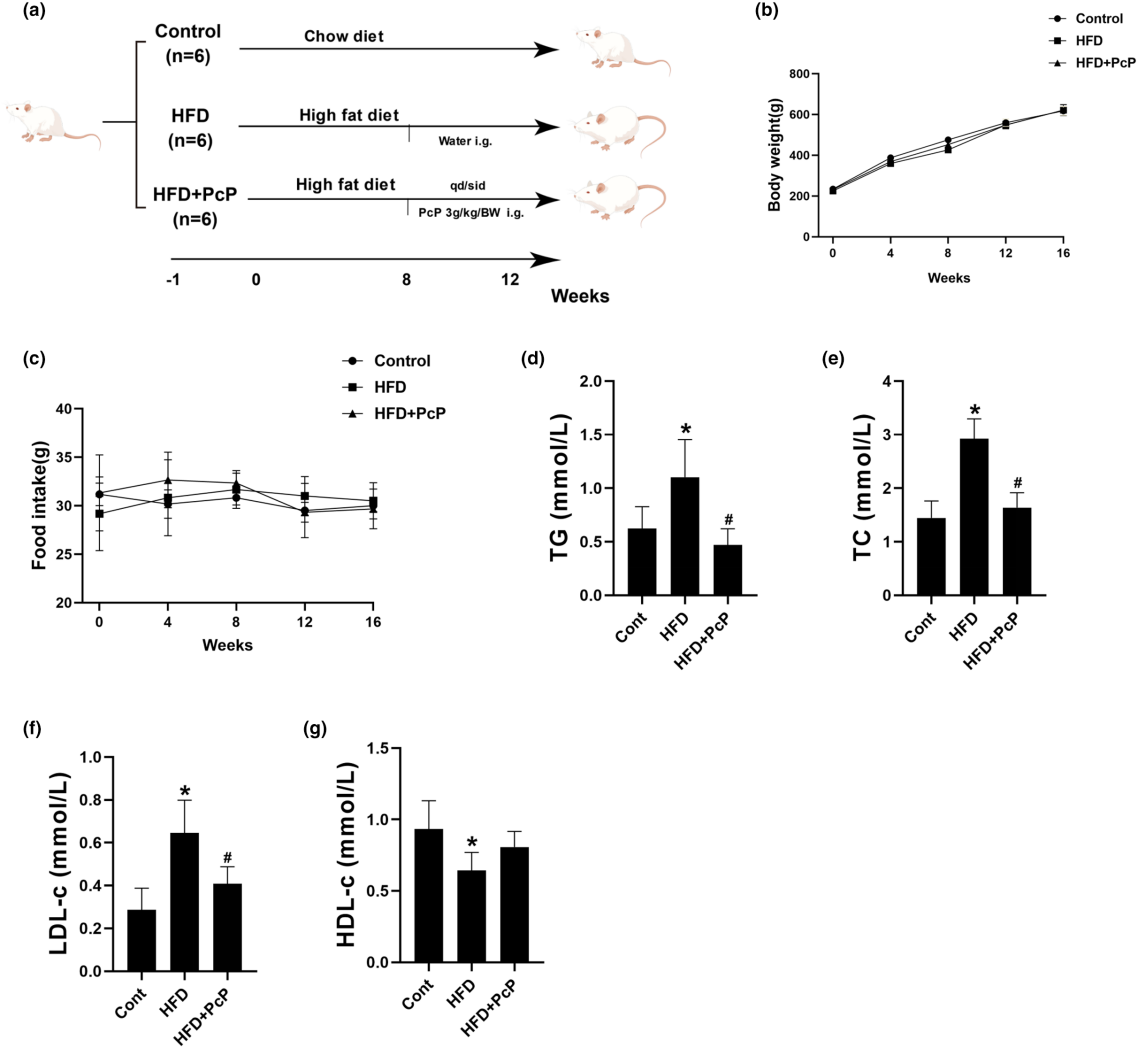
PcP extract attenuated dyslipidemia in HFD‐fed rats. (a) Schematic illustration of in vivo experiment. (b) The body weight curves of the rats in the different groups. (c) Mean food intake of the rats in the different groups. (d–g) Serum TG, TC, LDL‐c, and HDL‐c levels of rats in different groups. All experiments were repeated at least three times independently. The data are expressed as the mean ± SD (*p < .05 vs. the Cont group; ^#^ *p* < .05 vs. the HFD group).

**FIGURE 5:**
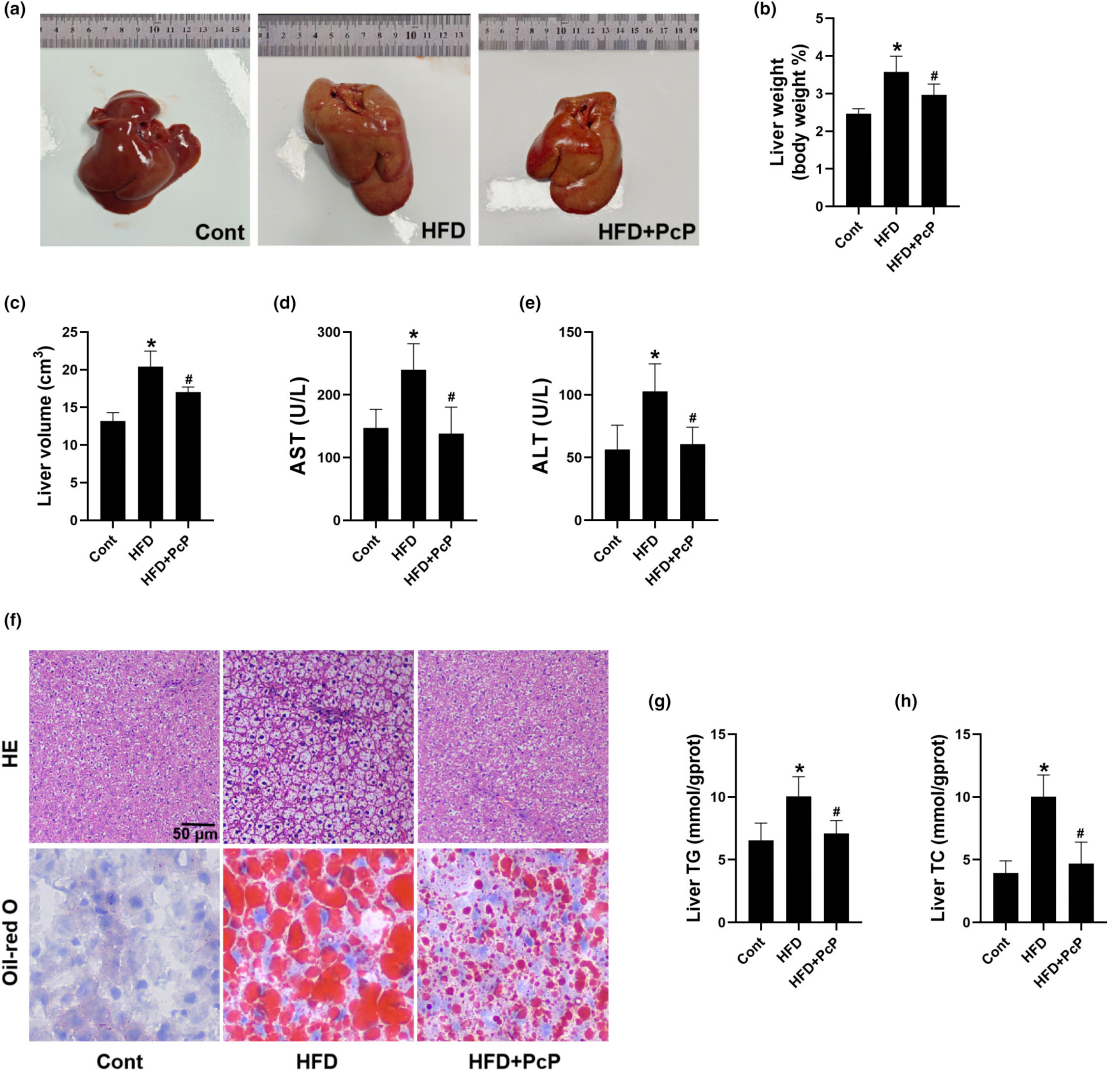
PcP extract attenuated hepatic injury in HFD‐fed rats. (a) Representative images of morphological changes in the livers of rats. (b, c) Changes in the liver weight and volume of rats in different groups. (d, e) Serum levels of ALT and AST in rats in different groups. (f) Representative images of HE and Oil Red O staining of liver tissues. (g, h) Total liver TG and TC contents. All experiments were repeated at least three times independently. The data are expressed as the mean ± SD (*p < .05 vs. the Cont group; ^#^ *p* < .05 vs. the HFD group).

To further confirm the above results in vitro, we orally administered PcP extracts to NAFLD rats once daily for 4 weeks at a dosage of 3 g/kg body weight (Figure 4a). Previously, we conducted a preliminary safety assessment of PcP in rats. The rats received PcP extracts at a dosage of 3 g/kg body weight (once a day for 4 weeks). We noted that the rats treated with PcP extracts did not exhibit significant changes in serum liver function or lipid metabolism indices (Figure S1). We next induced NAFLD in SD rats by feeding them an HFD as previously reported (Figure S2) (Li et al., 2021). The rats in the HFD group were then treated with or without PcP extract, and no noteworthy alterations in body weight or food intake were noted across the three groups (Figure 4b,c). Compared with those in the control group, the serum TG, TC, and LDL‐c levels were significantly increased, and the HDL‐c level was decreased. However, these changes were reversed in rats treated with PcP (*p* > .05 for HDL‐c, Figure 4d–g). Additionally, the livers of the rats in the HFD group appeared paler and fattier, and PcP treatment alleviated these changes (Figure 5a). Moreover, rats in the HFD group showed an increase in liver volume and weight, while these parameters decreased in the PcP group (Figure 5b,c). Additionally, the HFD‐fed rats exhibited elevated serum ALT and AST levels, which subsequently decreased following PcP supplementation (Figure 5d,e). We further conducted histopathological staining of liver tissues. HE staining revealed ballooning degeneration in hepatocytes and microvesicular steatosis in the livers of HFD‐fed rats, which was alleviated by the administration of PcP extract (Figure 5f). In addition, we noticed a significant accumulation of lipid droplets within the hepatocytes of rats in the HFD group, while PcP treatment effectively mitigated lipid deposition (Figure 5f). Additionally, PcP treatment notably decreased the liver TG and TC contents (Figure 5g,h). These results strongly suggest that the administration of PcP extract effectively attenuates dyslipidemia and hepatic injury in HFD‐fed rats.

### 3.6 PcP extract attenuated NLRP3/Caspase‐1/GSDMD pathway‐mediated hepatic pyroptosis in HFD‐fed rats

**FIGURE 6:**
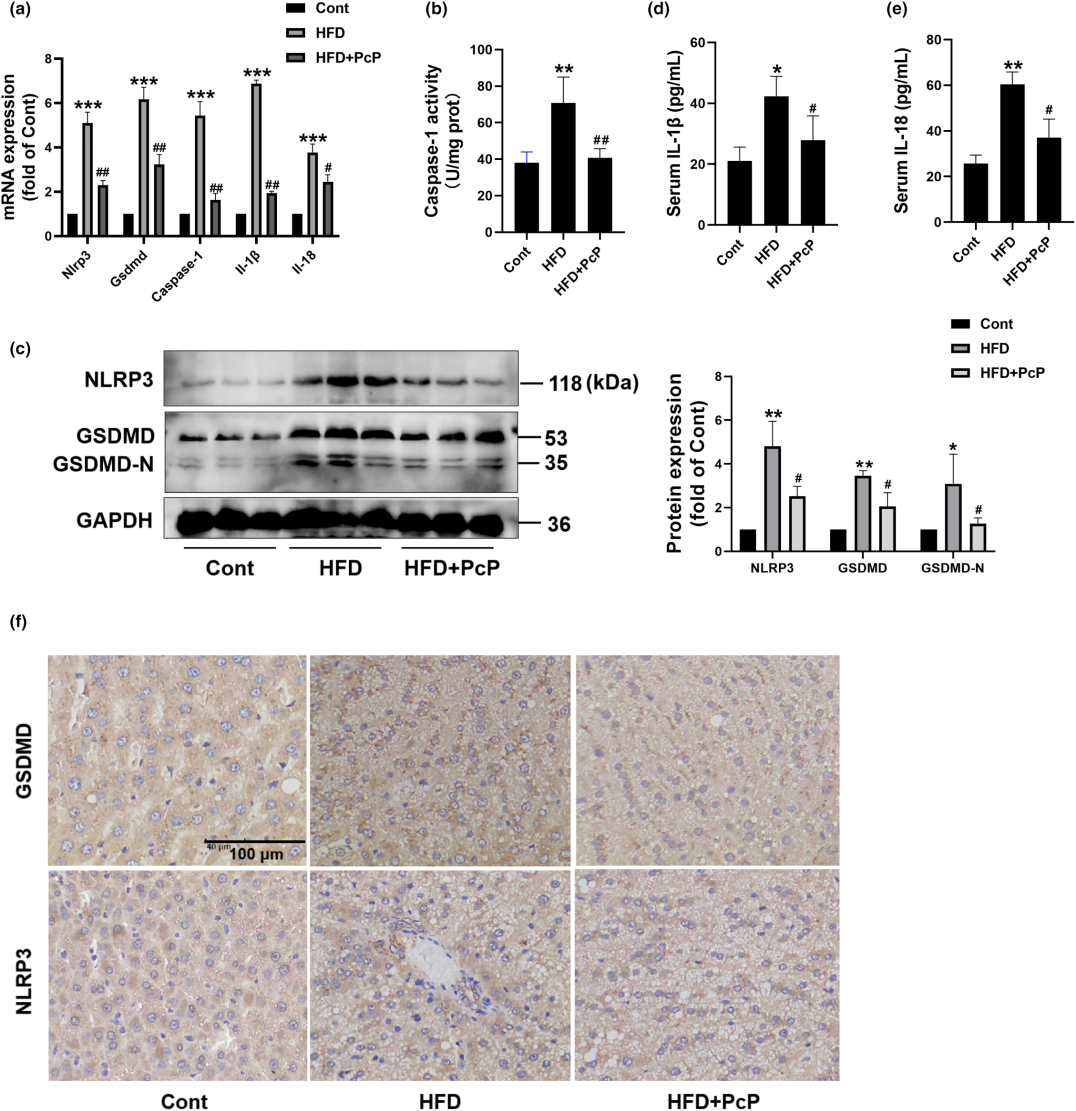
PcP extract attenuated NLRP3/Caspase‐1/GSDMD pathway‐mediated hepatic pyroptosis in HFD‐fed rats. (a) The mRNA levels of *Nlrp3*, *Caspase‐1*, *Gsdmd*, *Il‐1β*, and *Il‐18* in liver tissues were detected by Q‐PCR. (b) The caspase‐1 activity of liver tissues in different groups was measured by a caspase‐1 assay kit. (c) The protein levels of Nlrp3 and GSDMD/‐N in liver tissues were detected by Western blotting. (d, e) The serum levels of IL‐1β and IL‐18 were measured by ELISA. (f) Representative images of immunohistochemical staining of NLRP3 and GSDMD in liver tissues from the different groups. Scale bar, 100 μm. All experiments were repeated at least three times independently. The data are expressed as the mean ± SD (*p < .05, **p < .05, ***p < .01 vs. the Cont group; ^#^ *p* < .05, ^#*#*^ *p* < .01 vs. the HFD group).

The mRNA expression of *Nlrp3, Gsdmd, Caspase‐1, Il‐1β*, and *Il‐18* in liver tissues increased in rats in the HFD group and was subsequently abolished by PcP treatment (Figure 6a). Caspase‐1 activity and NLRP3 and GSDMD/‐N levels in liver tissues were elevated after HFD feeding, while these changes were reversed upon PcP supplementation (Figure 6b,c). Additionally, PcP treatment decreased the serum IL‐1β and IL‐18 levels (Figure 6d,e). Moreover, immunohistochemistry was performed to determine the localization of GSDMD and NLRP3 in liver tissues. We found that GSDMD and NLRP3 levels were greater in HFD‐fed rats than in control rats, while treatment with PcP effectively suppressed the levels of GSDMD and NLRP3 (Figure 6f). Collectively, these results indicate that PcP extract mitigates hepatic pyroptosis mediated by the NLRP3/caspase‐1/GSDMD pathway in HFD‐fed rats.

## 4 DISCUSSION

The etiology of NAFLD is multifactorial, encompassing an intricate interplay between genetic and metabolic elements. The current understanding is that numerous factors contribute to the development of NAFLD (Chi, 2017). Nevertheless, the primary cause of NAFLD is lipotoxicity resulting from the overabundance of fatty acids inside liver cells. Lipotoxicity contributes to hepatocyte death through the aforementioned pathways (Ibrahim et al., 2011). It has been suggested that mitigating hepatocyte death induced by lipotoxicity can effectively decelerate or potentially reverse the progression of NAFLD (Yang et al., 2022). Previous researchers have explored the importance of hepatocyte apoptosis in the progression of NAFLD (Feldstein & Gores, 2005). However, in our previous investigation, we employed palmitic acid to establish a lipotoxicity model in HepG2 cells, and the findings indicated that pyroptosis is the prevailing mode of cell death (Zeng et al., 2020). The activation of inflammasomes, particularly the NLRP3 inflammasome, predominantly initiates pyroptosis. In our study, we observed that inflammatory factors were robustly upregulated both in vivo and in vitro. We also found that Caspase‐1 was activated and that GSDMD/N and inflammatory cytokines were increased in cells and liver tissues, indicating that pyroptosis occurred. Previous research has shown that inhibiting NLRP3 can help mitigate the activation of pyroptosis (Singla et al., 2019), and we have previously suggested that inhibiting NLRP3‐mediated pyroptosis can ameliorate NAFLD in rats (Li et al., 2021). Based on these findings, we hypothesize that regulating the NLRP3‐mediated pyroptosis pathway is crucial for the treatment of NAFLD.

Managing NAFLD typically involves a comprehensive strategy that focuses on enhancing systemic metabolism and addressing body weight. Lifestyle modifications, including dietary adjustments and increased physical activity, are the preferred therapeutic interventions for NAFLD (Romero‐Gómez et al., 2017). However, the efficacy of these interventions is hindered by low patient adherence, rendering the treatment of NAFLD challenging (Porayko et al., 2022). Currently, there is no effective medication for the treatment of NAFLD. PcP, a traditional Chinese medicine, has been widely applied in clinical settings for the treatment of acute hepatitis and other liver diseases. Additionally, PcP is regarded as a functional food for the preservation of liver health and is often utilized as tea in China or to help individuals remain sober. Nevertheless, due to the intricate nature of traditional Chinese medicine, the precise mechanisms through which PcP exerts its hepatoprotective effects remain unclear. Cao et al. demonstrated that PcP extracts have a protective effect against chronic ethanol‐induced liver injury by alleviating oxidative stress and enhancing antioxidant defense systems (Cao et al., 2015). Additionally, several other studies have indicated that the hepatoprotective properties of PcP could be linked to the suppression of hepatic stellate cell activation and inflammation (Zhao et al., 2020; Zhou et al., 2018). However, limited research has been conducted on the effects of PcP on NAFLD. Xiaoxi Li reported that PcP extract attenuates NAFLD in mice by regulating the gut microbiota (Li, Zhao, et al., 2022). Nevertheless, the precise molecular mechanisms that underly this phenomenon remain unclear.

Herein, we simulated the human consumption of traditional Chinese medicine by administering a PcP extract solution to rats through oral gavage for 4 weeks. As anticipated, PcP extract demonstrated significant effectiveness in improving NAFLD and dyslipidemia in rats fed an HFD. Interestingly, an HFD significantly increased hepatic steatosis but did not lead to a significant increase in body weight in rats, which is consistent with our previous research. The results of many other studies also corroborate our findings. However, some studies have shown that body weight is significantly greater in rats fed an HFD. The main reason for this discrepancy may be the difference in rat strains and feed composition. The rats used in the experiment were in a rapid growth period because a reasonable nutritional ratio was provided, which allows for full absorption and utilization of nutrients and thus rapid growth. However, due to the increase in saturated fatty acids and sugars, an HFD can cause lipid metabolism disorders in rats, which affects the liver's metabolism of fatty acids and leads to fat accumulation. The liver is a digestive and biosynthetic organ, and when liver function is impaired in rats, it can affect their ability to synthesize and utilize other substances, thereby affecting weight gain. Additionally, we observed that treatment with PcP extract alleviated pyroptosis in rats with NAFLD, as evidenced by a reduction in caspase‐1 activity and NLRP3, GSDMD/N, IL‐1β, and IL‐18 levels. Mechanistically, we demonstrated that a specific NLRP3 agonist promotes the lysis of GSDMD, resulting in an increase in GSDMD‐N in vitro. This process is accompanied by augmented caspase‐1 activity and a concurrent increase in IL‐1β and IL‐18. Intriguingly, these protective effects were attenuated after nigericin was administered, suggesting that the NLRP3/GSDMD‐mediated pyroptosis pathway plays a significant role in PcP‐mediated defense against NAFLD.

In conclusion, for the first time, we report that the administration of PcP extract effectively alleviates hepatic steatosis by inhibiting pyroptosis. Inhibition of the NLRP3/Caspase‐1/GSDMD pathway might be a crucial mechanism that underlies the therapeutic effects of PcP. This study helps reveal the regulatory mechanisms of PcP in NAFLD.

## AUTHOR CONTRIBUTIONS

**Ruixi Luo:** Conceptualization (lead); funding acquisition (equal); methodology (lead); writing – original draft (lead). **Yudie Hu:** Conceptualization (equal); methodology (equal). **La Wang:** Investigation (equal); software (equal). **Zunli Ke:** Investigation (equal); methodology (equal). **Wenjia Wang:** Project administration (equal); supervision (equal); validation (equal). **Ping Wang:** Formal analysis (supporting); project administration (equal); resources (equal). **Weiyi Tian:** Conceptualization (lead); funding acquisition (equal); project administration (equal); resources (equal); supervision (lead); writing – review and editing (lead).

## FUNDING INFORMATION

This study was supported by the Guizhou Provincial Basic Research Program (Natural Science).

(QKHJC‐ZK[2021] YIBAN345), the Science and Technology Foundation of Guizhou Health Commission ([2021]92), the Growth Foundation for Young Scientists of the Education Department of Guizhou Province (QJJ[2022]203), and the Academic New Seedling Cultivation Project of Guizhou University of Traditional Chinese Medicine ([2023]‐30).

## ETHICS APPROVAL AND CONSENT TO PARTICIPATE

This study was approved by the Institutional Animal Care and Use Committee of Guizhou University of Traditional Chinese Medicine (Ethics approval number: 20220058).

## Supporting information


Figure S1

Figure S2

Table S1

Table S2

## Data Availability

The data that support the findings of this study are available from the corresponding author upon reasonable request.
