# Supplementary figures and images for: Penthorum chinense Pursh extract ameliorates hepatic steatosis by suppressing pyroptosis via the NLRP3/Caspase‐1/GSDMD pathway
Source: Food Sci Nutr. 2024 Apr 16;12(7):5176–87. doi: 10.1002/fsn3.4165 (PMC11266916; doi:10.1002/fsn3.4165)

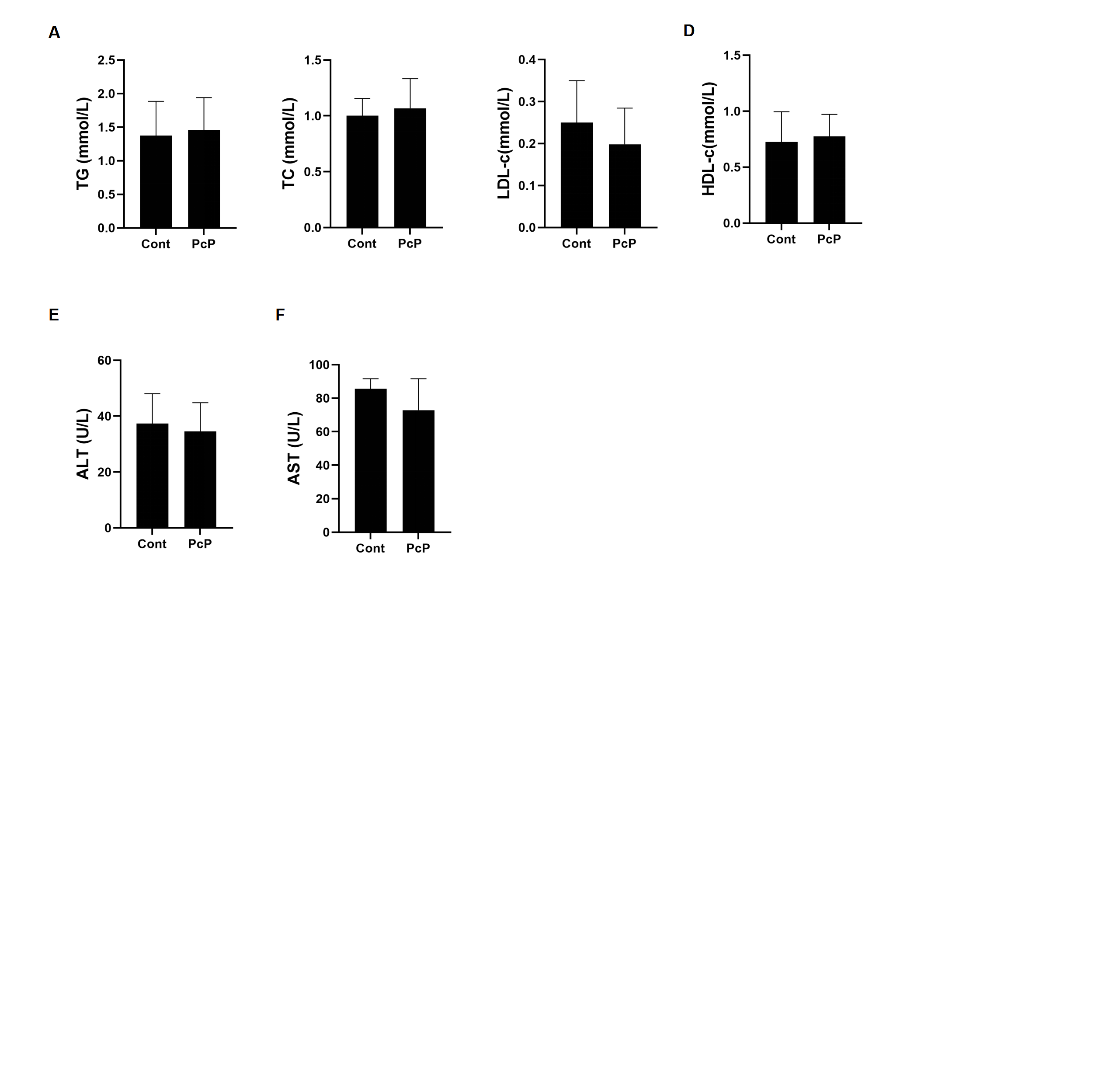

Supplement: Supplementary file 1 — Figure S1 [file FSN3-12-5176-s004.tif]

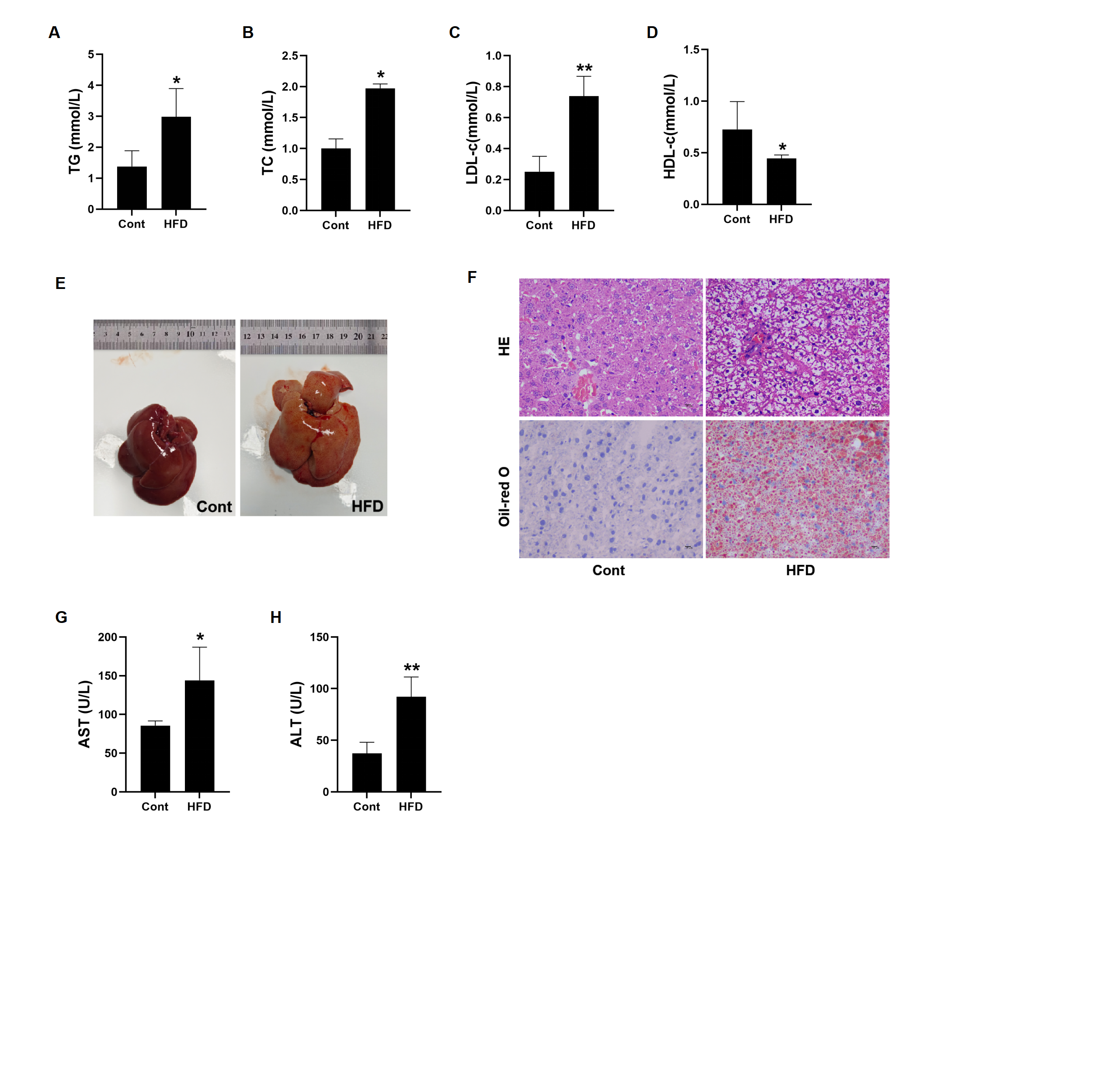

Supplement: Supplementary file 2 — Figure S2 [file FSN3-12-5176-s001.tif]
