# Supplementary material for: Penthorum chinense Pursh extract ameliorates hepatic steatosis by suppressing pyroptosis via the NLRP3/Caspase‐1/GSDMD pathway
Source: Food Sci Nutr. 2024 Apr 16;12(7):5176–87. doi: 10.1002/fsn3.4165 (PMC11266916; doi:10.1002/fsn3.4165)
Supplement: Supplementary file 3 — Table S1 [file FSN3-12-5176-s003.docx]

**TableS1: Primers used for Q-PCR**

| Gene | Forward | Reverse |
| --- | --- | --- |
| *Nlrp3* | GAGCTGGACCTCAGTGACAATGC | ACCAATGCGAGATCCTGACAACAC |
| *Gsdmd* | GTCTGCTTGCCGTACTCCATTCC | TGAAGAGCCTGCCTCCACCTC |
| *Il-1β* | GCCAACAAGTGGTATTCTCCA | TGCCGTCTTTCATCACACAG |
| *Il-18* | TGATAATGCTAGCGAACAGCCAACG | GGTCACCTAGTTAGTCCTCTTACTTC |
| *Caspase-1* | AGAGTCTGGAGCTGTGGCTACTG | ATGAGTGCTTGCCTGTGTTGGTC |

**Rat**

**Human**

| Gene | Forward | Reverse |
| --- | --- | --- |
| *NLRP3* | GGCAAATTCGAAAAGGGGTATT | CTGATTTGCTGAGAGATCTTGC |
| *GSDMD* | GCCAGAAGAAGACGGTCACCATC | TTCGCTCGTGGAACGCTTGTG |
| *IL-1β* | GCCAGTGAAATGATGGCTTATT | AGGAGCACTTCATCTGTTTAGG |
| *IL-18* | TGATATCGACCGAACAGCCAACG | GGTCACAGCCAGTCCTCTTACTTC |
| *CASPASE-1* | GATGATGATCACCGGTTTG | GAAGAAACACTCTGAGCAAGTC |
